# Long-term management of orbital Rosai–Dorfman disease with re-irradiation for locoregional recurrence: a case report

**DOI:** 10.3389/fonc.2026.1767610

**Published:** 2026-06-19

**Authors:** Sanjay Arvind Krishna, Anoop Remesan Nair, Hema Ashok Perumal, Malini Eapen, Pushpaja Kuttassery Ullattil, Debnarayan Dutta

**Affiliations:** 1 Department of Radiation Oncology, Amrita Institute of Medical Sciences and Research, Amrita Vishwa Vidyapeetham, Kochi, India; 2 Department of Pathology, Amrita Institute of Medical Sciences and Research, Amrita Vishwa Vidyapeetham, Kochi, India

**Keywords:** case report, optic neuropathy, orbital diseases, radiotherapy, re-irradiation, Rosai–Dorfman disease

## Abstract

**Introduction:**

Rosai–Dorfman–Destombes disease (RDD) is a rare non-Langerhans histiocytic disorder with occasional extranodal involvement. Orbital disease can mimic neoplastic or inflammatory conditions, leading to diagnostic and therapeutic challenges. Standardized management strategies remain undefined.

**Aim:**

To describe the diagnostic complexity and therapeutic role of radiation therapy, including re-irradiation, in orbital RDD.

**Case presentation:**

A 58-year-old male presented with nasal obstruction, later progressing to orbital involvement. Histopathology confirmed RDD (S100+, CD68+, CD1a–). Initial corticosteroid therapy provided partial relief. Image-guided radiotherapy (36 Gy in 20 fractions) achieved complete remission for five years. On recurrence with proptosis and compressive optic neuropathy, re-irradiation (24 Gy in 12 fractions) was delivered after careful review of prior dose constraints. The patient demonstrated improved visual acuity (6/60 to 6/36), stable MRI findings, and no late radiation toxicity at two-year follow-up.

**Conclusion:**

This case highlights the feasibility and efficacy of radiation therapy, including re-irradiation, in orbital RDD when medical management fails. Re-irradiation achieved durable local control, preserved visual function, and adds to the limited evidence supporting its role in recurrent disease.

## Introduction

Rosai–Dorfman–Destombes disease (RDD), first described by Destombes in 1965 (1), is a rare non-Langerhans cell histiocytosis characterized by the accumulation of activated histiocytes in nodal and extranodal sites (2). Although often self-limiting, outcomes vary depending on disease location and extent. Extranodal involvement occurs in up to 43% of cases, with orbital disease representing an uncommon but clinically significant presentation (3). Histopathological evaluation revealed emperipolesis with immunohistochemical positivity for S100 and CD68 and negativity for CD1a, confirming the diagnosis of Rosai–Dorfman disease. This immunophenotypic profile also facilitated exclusion of important differentials, including Langerhans cell histiocytosis (characteristically CD1a positive), IgG4-related disease (associated with IgG4-positive plasma cells and fibrosis), and Erdheim–Chester disease, which typically demonstrates foamy histiocytes along with a distinct clinicoradiological pattern (4, 5).

## Case presentation

A 58-year-old male presented with nasal obstruction and shortness of breath. Nasal endoscopy revealed a fleshy mass occupying the right nasal cavity. Histopathology demonstrated emperipolesis and immunopositivity for S100 and CD68, consistent with RDD (**Figure 1**). Baseline CT thorax with abdomen screening was done as part of baseline workup before initiating any treatment to rule out other foci of the disease.

**Figure 1:**
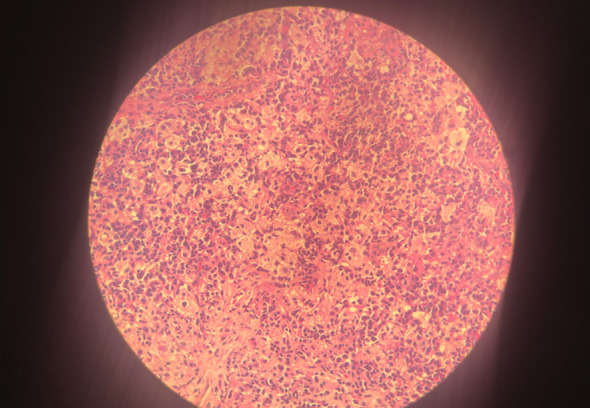
Pathological Slide of RDD which shows histiocyte collections with emperipolesis amidst inflammation predominant in plasma cells.

Initial management with oral prednisolone (50 mg daily, tapered over six months) provided partial relief. One year later, the patient developed right eye proptosis and visual disturbance. MRI revealed a 2.0 × 1.76 cm enhancing intraconal orbital lesion (**Figure 2**). Low-dose steroids were re-initiated.

**Figure 2:**
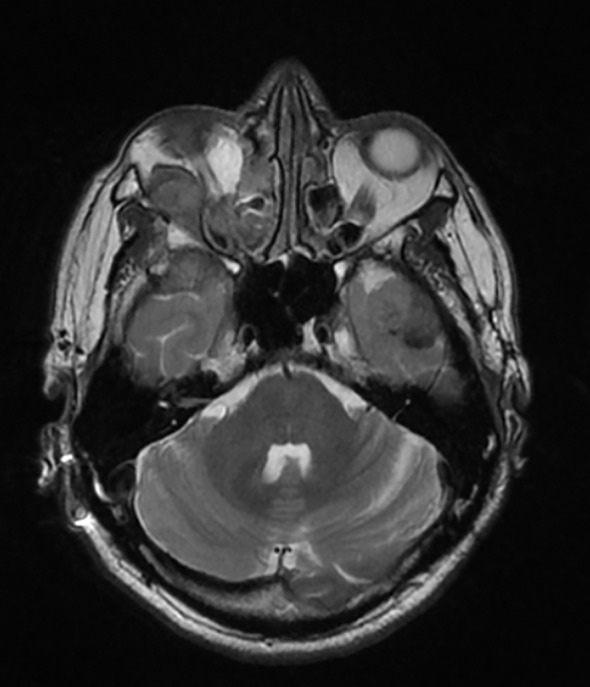
Pre-treatment MRI brain with contrast (Axial T2 FSE).

Two years later, disease progression was confirmed on MRI. Image-guided radiotherapy (IGRT) was delivered to the right orbit, nasal cavity, and nasopharynx (36 Gy in 20 fractions), achieving complete remission for five years (**Table 1**).

**Table 1:** Sequence of events.

Year	Event
2015	Nasal mass → biopsy for Initial Diagnosis
2016	Orbital involvement
2017	External Beam Radiation Therapy 36 Gy
2017–2022	Remission – Under regular MRI follow up
2023	Recurrence -- Re-RT 24 Gy
2022-2025	Stable with serial Annual MRI follow up

On Follow up, He presented with worsening proptosis and compressive optic neuropathy (**Figure 3**), Additionally a site specific FDG PET scan of the brain (**Figures 4** and **5**) was done to delineate the gross disease and help in radiation treatment planning. Re-irradiation was planned after reviewing prior dose constraints (**Table 2**) in detail. IGRT (Image guided Radiation therapy) was delivered to the orbit and nasal cavity (24 Gy in 12 fractions). At two-year follow-up, MRI showed stable disease, visual acuity improved from 6/60 to 6/36, and no late radiation toxicity was observed.

**Figure 3:**
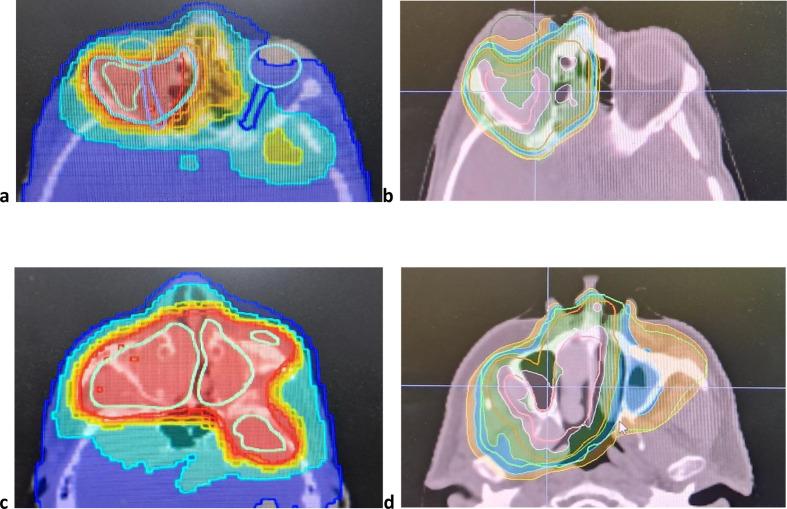
Dose profile comparison [initial plan – **(a, c)** and re-RT plan **(b, d)**].

**Figure 4:**
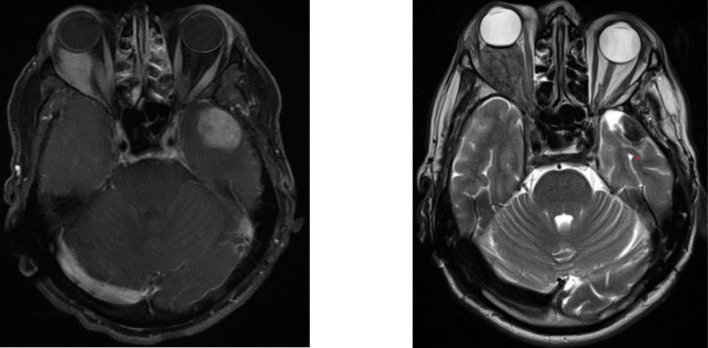
Post Treatment MRI Brain with contrast [ Axial T1 contrast sequence] Enhancing soft tissue lesion in the right orbit intra conal fat seen that abuts the lateral rectus and inferior rectus and now increased in size also involving the PNS. Extra axial lesion in left temporal region 2.4x1.4cms seen remained status quo.

**Table 2:** Dosimetric parameters.

Structure	Dose (2017)	Dose (2023) – ReRT
Dose/Fractions	36 Gy in 20 Fractions	24 Gy in 12 Fractions
GTV (Dmax)	38.7 Gy	25.5 Gy
Right Optic Nerve (Dmax)	37.7 Gy	23.2 Gy
Left Optic Nerve(Dmax)	33.1 Gy	13.7 Gy
Right Eye Ball (Dmax)	37.7 Gy	24.2 Gy
Left Eye Ball (Dmax)	30.3 Gy	13.7 Gy
Brainstem (Dmax)	24.1 Gy	13.1 Gy
Left Parotid (Dmean)	0.25 Gy	5.1 Gy
Right Parotid (Dmean)	4.97 Gy	5.4 Gy

**Figure 5:**
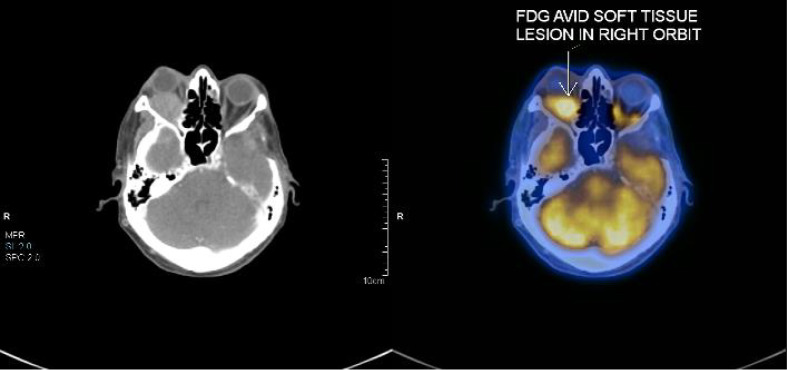
Recurrence – FDG PET brain showing right orbit intraconal soft tissue lesion of size 3 x 2.5 x 5 cms causing proptosis posteriorly extending up to orbital apex & inferiorly up to Infratemporal fossa. Laterally minimal extension to lateral rectus muscle.

## Discussion

Orbital Rosai–Dorfman–Destombes disease (RDD) is an uncommon manifestation of a rare histiocytic disorder, accounting for approximately 11% of extranodal cases (4). An essential component in diagnosing RDD is distinguishing it from other histiocytic and inflammatory conditions that may present with overlapping clinical and radiological features. Langerhans cell histiocytosis is usually identified by expression of CD1a and Langerin, both of which were absent in the present case. IgG4-related disease, an important differential in orbital pathology, is characterized by dense lymphoplasmacytic infiltration, increased IgG4-positive plasma cells, and storiform fibrosis—features not observed in this patient. Erdheim–Chester disease, although also CD68 positive, is typified by foamy histiocytes, frequent BRAF mutations, and a characteristic pattern of multisystem involvement. In contrast, the presence of emperipolesis together with S100 and CD68 positivity and CD1a negativity strongly supports the diagnosis of RDD. Careful recognition of these distinguishing histopathological and immunophenotypic features is crucial, as therapeutic approaches and prognosis differ significantly among these entities. Misdiagnosis has been reported, including cases initially mistaken for uveal melanoma (6) or IgG4-related disease (7), underscoring the importance of pathological expertise and multidisciplinary evaluation.

### Diagnostic considerations

RDD was first described by Destombes in 1965 (1) and later characterized by Foucar et al. (2). Extranodal involvement is seen in up to 43% of patients (3), with orbital disease being particularly rare but clinically significant. Histopathology remains the gold standard, though radiological features such as well-defined enhancing orbital masses on MRI can support suspicion. Ancillary testing is often required to exclude mimics such as Erdheim–Chester disease (8), Langerhans cell histiocytosis, or IgG4-related disease. Awareness of these differential diagnoses is critical, as treatment strategies differ substantially.

### Surgical management

Surgical excision is frequently considered for localized orbital lesions. Several reports demonstrate durable control following complete or partial excision (9–13). Yataco-Vicente et al. (10) described a patient with orbital RDD successfully managed with surgery alone, while Al-Maghrabi et al. (11) reported long-term control after excision. However, complete resection may be challenging when lesions are adjacent to critical structures such as the optic nerve or extraocular muscles. In such cases, surgery carries significant risk of morbidity, including vision loss or diplopia. Our case highlights this limitation, as surgical intervention was not feasible given the lesion’s proximity to the optic nerve.

### Role of radiotherapy

Radiotherapy has emerged as a valuable option in orbital RDD, particularly when surgery is not feasible or recurrence occurs. Vemuganti et al. (14) reported favorable outcomes with EBRT in orbital disease, while Cooper et al. (15) described a pediatric patient achieving durable control after radiotherapy. Virk et al. (16) demonstrated the utility of RT in a case presenting as optic neuritis. Case series in cutaneous RDD also support RT as a safe and effective modality (17). Importantly, the doses required for disease control are relatively low compared with malignant tumors, typically ranging from 12–30 Gy, making cumulative toxicity more manageable.

Our case adds to this evidence by demonstrating the feasibility of re-irradiation. Following initial RT (36 Gy in 20 fractions), the patient remained progression-free for five years. On recurrence, re-irradiation (24 Gy in 12 fractions) achieved durable local control and functional preservation, with improved visual acuity and no late toxicity at two-year follow-up. This experience highlights that re-irradiation can be considered in carefully selected patients, particularly when cumulative doses remain within tolerance limits for critical structures such as the optic nerve and retina.

### Systemic therapies

Systemic therapy is generally reserved for refractory or disseminated disease. Corticosteroids remain the most frequently employed, but responses are variable and often transient (18, 19). Immunomodulatory agents, chemotherapy, and targeted therapies have been explored. Diamond et al. (20) demonstrated efficacy of MEK inhibition in histiocytic neoplasms, while López-Aldabe et al. (21) reported successful use of cobimetinib in drug-refractory RDD. Pulsoni et al. (22) described corticosteroid and chemotherapy use in nodal disease. Mercaptopurine has been reported in orbital and intracranial RDD (7), and rituximab has been used in selected cases. Consensus guidelines recommend systemic therapy for unresectable disease, with imaging surveillance every 6–12 months (4). Nevertheless, the rarity of orbital RDD limits the availability of standardized protocols, and most treatment decisions remain individualized.

### Broader literature context

Several case reports and series enrich our understanding of orbital RDD. Su et al. (23) described orbital RDD managed with surgery and adjuvant RT. Masoomian et al. (24) reported ophthalmic manifestations in five patients, emphasizing the heterogeneity of presentation. Gaitonde (25) provided an overview of extranodal RDD, while Razanamahery et al. (8) discussed overlap with Erdheim–Chester disease, emphasizing the need for molecular characterization. Additional reports have described sinonasal involvement, cutaneous and ocular manifestations, and recurrent nodal disease in RDD, further highlighting the heterogeneity of presentation and management approaches (26–28).

### Novelty of our case

Our case is unique in demonstrating safe and effective re-irradiation in orbital RDD. While radiotherapy has been reported previously (14–16), re-irradiation has rarely been documented. The relatively low dose requirements for RDD compared with malignant tumors make re-irradiation feasible, provided careful attention is paid to cumulative dose constraints. In our patient, re-irradiation achieved durable local control, preserved visual function, and avoided late toxicity. This adds to the limited literature supporting radiotherapy as a viable option in recurrent orbital RDD.

**Review Of literature –** (**Table 3**).

**Table 3:** Comparison of our case with existing literature on orbital Rosai–Dorfman disease.

S. no	Authors/year	No. of patients	Age (years)	Gender	Clinical features	Site	Visual acuity	Lesion size (cm)	Imaging	Treatment	Radiation dose	Follow-up (months)	Recurrence
1	Xin Su et al., 2019 (23)	1	45	Male	Proptosis	Left eye	Not available	3.7	MRI	Surgery → Adjuvant RT	20 Gy in 10 fractions	12	None
2	Yataco et al., 2018 (10)	1	42	Male	Proptosis	Right eye	Preserved	5.1	CT	Surgery	Not applicable	4	None
3	Arun et al., 2009–2011 (9)	1	60	Female	Proptosis	Left eye	Not available	5	MRI	Surgery	Not applicable	Not available	No
4	Valentino et al., 2016 (7)	1	70	Male	Diminution of vision	Left eye + intracranial extension	Decreased (right eye)	Not available	MRI	Chemotherapy	Not applicable	24	None
5	Al-Maghrabi et al., 2019 (11)	1	53	Male	Diminution of vision	Right eye	Decreased (left eye)	4.5	MRI	Surgery	Not applicable	24	None
6	Masoomian et al., 2020 (24)	5	Mean 33	3 Male, 2 Female	Diminution of vision	Bilateral	Decreased	Not available	CT/MRI	Surgery	Not applicable	24	None
7	Yousef et al., 2018 (6)	1	18	Female	Loss of vision	Right eye	Counting fingers only	1.7	MRI	Surgery	Not applicable	Not available	None
8	Vemuganti et al., 2008 (14)	5	Mean 65	Not specified	Proptosis	Bilateral	Preserved	Not available	Not available	Surgery	Not applicable	18	None
9	Renato et al., 2010 (9)	1	14	Female	Fleshy orbital mass	Right eye	Preserved	Not available	Ultrasound	Surgery	Not applicable	24	None
10	Cooper et al., 2008 (15)	1	12	Female	Pain	Left eye	Decreased	Not available	MRI	Steroids → Surgery → Chemotherapy → RT	12 Gy in 6 fractions	7	None
11	Iyengar et al., 2020 (5)	1	17	Male	Swelling	Left eye	Not available	Not available	MRI	Steroids → Surgery → Monoclonal antibody → Surgery	Not applicable	Not available	Progression
12	Our study, 2025	1	58	Male	Proptosis	Right eye	Preserved	2.0	MRI	Steroids → RT → Re-RT	36 Gy in 20 fractions + 24 Gy in 12 fractions	84	5 years progression-free survival

## Data Availability

The original contributions presented in the study are included in the article/supplementary material. Further inquiries can be directed to the corresponding author.
